# Improved Brain Pathology and Progressive Peripheral Neuropathy in a 15 Year Old Survivor of Infantile Krabbe Disease Treated With Umbilical Cord Transplantation

**DOI:** 10.3389/fnmol.2022.888231

**Published:** 2022-07-28

**Authors:** Julia Kofler, Maria L. Beltran-Quintero, Anne Rugari, Giulio Zuccoli, Sarah Klotz, Maria L. Escolar

**Affiliations:** ^1^Division of Neuropathology, Department of Pathology, University of Pittsburgh, Pittsburgh, PA, United States; ^2^Program for the Study of Neurodevelopment in Rare Disorders, UPMC Children’s Hospital of Pittsburgh, University of Pittsburgh Medical Center, Pittsburgh, PA, United States; ^3^Partners for Krabbe Research, Cincinnati, OH, United States

**Keywords:** Krabbe disease, umbilical cord transplantation, globoid cell leukodystrophy, neurodegeneration, peripheral neuropathy

## Abstract

**Objective:**

Krabbe disease is a fatal leukodystrophy caused by deficiency in galactocerebrosidase enzyme activity. The only currently available therapy is hematopoietic stem cell transplantation with bone marrow or umbilical cord blood (UCBT), which leads to increased lifespan and functional abilities when performed in the preclinical stage. While stabilization of white matter disease has been seen on serial MRI studies, neuropathological changes following transplantation have not been documented so far.

**Materials and Methods:**

We report the first postmortem examination of a 15-year-old female patient with infantile Krabbe disease after UCBT in infancy.

**Results:**

In contrast to an untreated Krabbe disease brain, which showed severe myelin and oligodendrocyte loss with occasional globoid cells, the transplanted brain displayed markedly improved myelin preservation, but not reaching normal myelination levels. Consistent with the transplanted patient’s clinical presentation of pronounced deficits in gross motor skills, corticospinal tracts were most severely affected. No globoid cells or evidence of active demyelination were observed in the central nervous system, indicative of at least partially successful functional restoration. This was corroborated by the identification of male donor-derived cells in the brain by *in situ* hybridization. Unlike the observed disease stabilization in the central nervous system, the patient experienced progressive peripheral neuropathy. While diminished macrophage infiltration was seen postmortem, peripheral nerves exhibited edema, myelin and axon loss and persistent Schwann cell ultrastructural inclusions.

**Conclusion:**

Umbilical cord blood transplantation was able to alter the natural disease progression in the central but less so in the peripheral nervous system, possibly due to limited cross-correction of Schwann cells.

## Introduction

Krabbe disease, or globoid cell leukodystrophy, is a neurodegenerative disorder, primarily involving the white matter in the peripheral (PNS) and central nervous system (CNS). It is an autosomal recessive disease caused by various mutations in the galactocerebrosidase (*GalC*) gene. During myelin turnover, the decreased or lost GALC enzyme activity leads to impaired degradation of its two main substrates, galactosylceramide (galactocerebroside) and galactosylsphingosine (psychosine) (Wenger et al., 2001; Suzuki, 2003; Graziano and Cardile, 2015). The accumulation of cytotoxic psychosine results in the apoptosis of myelin-forming cells and induction of inflammatory processes, forming the basis of the “psychosine hypothesis” of Krabbe disease (Miyatake and Suzuki, 1972; Suzuki, 1998). The impaired degradation of galactosylceramide leads to the characteristic formation of enlarged, spherical and sometimes multinucleated macrophages, called globoid cells, in the white matter of the CNS (Suzuki, 2003; Graziano and Cardile, 2015). More recent evidence suggests that psychosine is also detrimental to other cell types in the brain, leading to early microglial and astrocyte activation and initiation of an inflammatory cascade, which in turn may contribute to demyelination (Karumuthil-Melethil and Gray, 2016; Nicaise et al., 2016; Potter and Petryniak, 2016).

The disease is typically subdivided into four groups based on age-of-onset of clinical symptoms (Wenger et al., 2001; Suzuki, 2003). The most common form is the early infantile presentation, with an estimated incidence of 1 in 100,000 to ∼400,000 (Orsini et al., 2016). Recently, our group has proposed reclassification of Krabbe variants according to our prospective natural history studies (Bascou et al., 2018; Beltran-Quintero et al., 2019), eliminating the Early Infantile phenotype in favor of the Infantile phenotype which now encompasses patients that present between 0 and 12 months of age. The Late-Infantile phenotype now includes patients with symptom onset from 13 to 36 months. The other two phenotypes described for Krabbe disease are juvenile (onset of symptoms from 3 to 16 years) and adult forms (onset in patients > 16 years old).

Pathology in Krabbe disease is characterized by a rapid and nearly complete disappearance of myelin and myelin-forming cells, i.e., the oligodendrocytes in the CNS and the Schwann cells in the PNS, associated with reactive astrocytic gliosis, and accumulation of globoid cells that contain strongly periodic acid-Schiff (PAS)-positive inclusions, biochemically identical with galactosylceramide (Suzuki, 2003; Suzuki and Vanier, 2004; Walkley et al., 2015). Peripheral nerves exhibit endoneurial fibrosis and segmental demyelination with tubular inclusions in endoneurial macrophages and Schwann cells (Suzuki, 2003).

Hematopoietic stem cell transplantation, most commonly in the form of umbilical cord blood transplantation (UCBT), is currently the only available treatment modality that has shown to favorably alter the disease course by improving lifespan and functional abilities, but only if performed before onset of clinical symptoms (Krivit et al., 1998; Escolar et al., 2005; Sakai, 2009; Wright et al., 2017; Allewelt et al., 2018). Functional outcomes in pre-symptomatic or minimally symptomatic patients depend heavily on disease burden at the time of HSCT (Wright et al., 2017). Serial brain magnetic resonance imaging (MRI) studies of transplanted Krabbe patients revealed stabilization of CNS disease in the vast majority of subjects, but nerve conduction studies demonstrated only transient improvements of nerve conduction velocities (Wright et al., 2017). The rationale for this therapeutic approach is that bone marrow-derived macrophages secrete the missing protein, which then leads to cross-correction of the enzymatic defect in myelin-forming cells (Wenger et al., 2000; Graziano and Cardile, 2015). However, this mechanism has recently been called into question by two studies failing to identify convincing enzymatic restoration in target cells in human metachromatic leukodystrophy brains (Wolf et al., 2020) and Krabbe disease peripheral nerves (Weinstock et al., 2020) despite clear evidence of donor-derived macrophage engraftment and improved myelination. Both studies propose restoration of phagocytic response and modulation of neuroinflammation by donor macrophages as alternative mechanisms.

Aside from the above report on peripheral nerves from a transplanted Krabbe disease case, information about neuropathologic changes following transplantation is limited to animal models of Krabbe disease. In murine models, bone marrow transplantation resulted in extended life spans, increased GalC activity and less psychosine accumulation in the nervous system (Yeager et al., 1984; Ichioka et al., 1987; Hoogerbrugge et al., 1988a; Luzi et al., 2005). This was accompanied by increased numbers of remyelinated fibers and significantly decreased numbers of the typical inclusion-laden macrophages in peripheral nerves, but inclusions were still seen in some remyelinating Schwann cells (Yeager et al., 1984; Kondo et al., 1988; Luzi et al., 2005). In the central nervous system, improvements were only appreciated after prolonged survival and consisted of evidence of remyelination and disappearance of inclusion-bearing macrophages (Yeager et al., 1984; Suzuki et al., 1988; Luzi et al., 2005). Similar to peripheral Schwann cells, inclusions were still identified in oligodendrocytes. Taken together, these studies suggest that bone marrow transplantation was beneficial, but that the metabolic defect was only partially corrected. To date there are no pathologic studies on UBCT treated patients. This study aims to describe the neuropathological changes seen in humans, comparing a patient that progressed untreated to one that was treated with UCBT before the onset of symptoms. This study was made possible by the Program for the Study of Neurodevelopment in Rare Disorders (NDRD), which established the NDRD Brain and Tissue Bank at the University of Pittsburgh with a focus on pediatric neurodegenerative diseases. This report is the first histopathological characterization of the effects of UBCT treatment on the degenerative changes associated with Krabbe disease.

## Materials and Methods

### Ethics Approvals and Patient Consents

Clinical studies were approved by the institutional review boards at the University of Pittsburgh (PRO11050036) and University of North Carolina, Chapel Hill (UNC-CH 08-0237). Informed consent was obtained for all patients evaluated at the NDRD at the University of Pittsburgh. Patients evaluated at UNC-CH either gave informed consent or were included under a waiver granted by UNC-CH. The parents of two patients with Infantile Krabbe disease consented to autopsy and tissue donation to the University of Pittsburgh NDRD Brain and Tissue Bank. All banking procedures were approved by the Committee for Oversight of Research and Clinical Training Involving Decedents (CORID) at the University of Pittsburgh.

### Clinical History Review

Medical records of both patients were reviewed. Patient 1 was participant in a longitudinal natural history study of Krabbe disease (Beltran-Quintero et al., 2019). Patient 2 was participant in a long-term study evaluating the outcome of UCBT in infancy (Escolar et al., 2005; Wright et al., 2017). In both studies, patients had prospective standardized evaluations over their lifetime. Patients were asked to return for follow up every 3 months for the first year, every 6 months during the second year and then every year. At each visit, a detailed history was obtained and a physical examination and a battery of neurodevelopmental tests were performed as previously described in detail (Escolar et al., 2005; Wright et al., 2017; Beltran-Quintero et al., 2019). Chronological age was compared to developmental age as determined by standardized testing using the Mullen Scales of Early Learning (Mullen, 1995) and the Peabody Developmental Motor Scales (Folio and Fewell, 2000). Nerve conduction studies, MRI and other electrophysiological studies were scheduled yearly. Outcomes were compared to the norms of typically developing children.

### Neuropathology Assessments

Brain and tissue banking procedures followed a standardized protocol. After extraction, the brain was bisected by a mid-sagittal cut. One hemisphere was serially sectioned in the coronal plane and frozen at −80°C; the other hemisphere was fixed in formalin for about 2 weeks and then serially sectioned in the coronal plane. The spinal cord was removed from cervical to Cauda Equina and multiple cross-sections from cervical, thoracic, and lumbar levels were either frozen or fixed in formalin. Segments of femoral nerve and brachial plexus were collected and either frozen or fixed in formalin or glutaraldehyde. Representative sections of formalin-fixed cortex and white matter of all four lobes, subcortical gray matter, brainstem, cerebellum, peripheral nerves and cervical, thoracic and lumbar spinal cord were sampled for histologic examination. All sections were stained with hematoxylin and eosin (H&E) to assess neuronal loss, white matter rarefaction, reactive astrogliosis and macrophage/globoid cell infiltrates. Luxol Fast Blue (LFB) stains were performed on all sections to assess myelination status, with cresyl violet (CV) or periodic acid Schiff (PAS) as counterstain. Immunohistochemical stains for CD68 (clone KP1, cat# M0814, 1:500, pH 6.0 citrate antigen retrieval; Dako, Carpinteria, CA, United States) and neurofilament (clone 2F11, cat# M0762, 1:50, pH 6.0 citrate antigen retrieval; Dako) were done on select sections using standard protocols.

For ultrastructural studies, tissue samples were fixed in glutaraldehyde followed by incubation in 1% osmium tetroxide for 1 h at room temperature. Samples were rinsed and dehydrated in increasing ethanol concentrations (50, 70, and 100%), followed by incubation in propylene oxide and embedding in resin. Semithin sections were cut at 1 μm thickness and stained with toluidine blue. Thin sections (60–90 nm) for electron microscopic examination were stained with aqueous uranyl acetate and lead citrate.

DNA fluorescence *in situ* hybridization (FISH) was performed with X and Y chromosome probes (Vysis CEP X SpectrumOrange/Y SpectrumGreen DNA probe kit, Abbott, Des Plaines, IL, United States) following the manufacturer’s instructions to detect male donor-derived cells in the brain of the female transplanted patient. RNA ISH studies were performed on formalin-fixed paraffin-embedded tissue sections of the brain and peripheral organs using a cocktail of three RNAscope Target Probes for Y chromosome specific genes (EIF1AY, ZFY, and UTY; Advanced Cell Diagnostics, Hayward, CA, United States). Pretreatment, hybridization and detection techniques were performed according to manufacturer’s protocols.

## Results

### Clinical Case Histories

Using the newest classification system, patient 1, a male, was diagnosed at 9 months of age following several months of progressive clinical symptoms and signs, starting at 6 months of age with changes in muscle tone and stiffness in his lower extremities. At 7 months, he lost his ability to hold his head up and to reach for objects. His GALC enzyme level was markedly decreased at 0.06 nmol/h/mg of protein and his *GALC* mutation analysis showed homozygosity for the 30 kb deletion of the C-terminal end of the *GALC* gene establishing a diagnosis of Krabbe disease. This mutation is known to be predictive of the infantile phenotype (Wenger et al., 2014). During his first assessment, he was found to be in stage II of disease progression (Escolar et al., 2006), which made him not eligible for UCBT.

**FIGURE 1:**
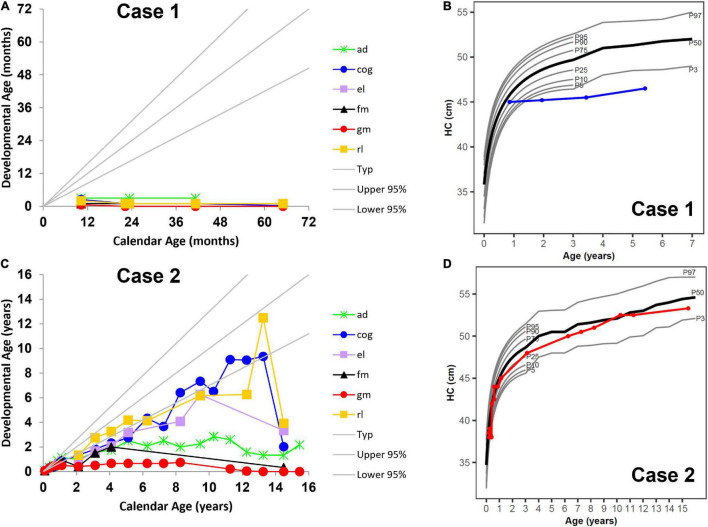
Neurocognitive development across multiple domains **(A)** without umbilical cord blood transplantation (UCBT) (case 1) and **(C)** after UCBT (case 2) using age-equivalent scores (i.e., developmental age). Head circumference (HC) development **(B)** without UCBT (case 1) and **(D)** after UCBT (case 2). The colored lines in panels **(A,C)** represent individual domains. The colored lines in panels **(B,D)** represent case 1 (blue) and case 2 (red). In all panels, the area between the gray lines represents typical development. ad, adaptive behavior; cog, cognitive; el, expressive language; fm, fine motor; gm, gross motor; rl, receptive language. P3, P5, P10, P25, P50, P75, P90, P95, and P97 represent percentiles (P).

In line with published natural disease progression (Bascou et al., 2018), longitudinal developmental testing showed severe disability with rapid progression and loss of function in all areas of development reverting to that of a 1-month-old (Figure 1A). His growth parameters showed stunting of head growth and microcephaly (Figure 1B), while height and weight remained in the normal range at the 25th percentile, with caloric intake coming from gastrostomy tube feeding. At his last visit at 5 years 5 months, he had progressed to stage IV of the disease and had developed seizures, temperature instability and was having multiple apneic episodes.

**FIGURE 2:**
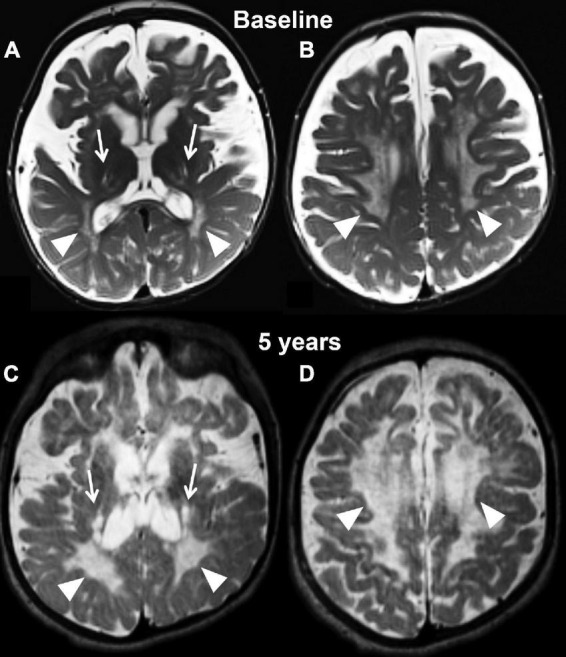
Axial T2 weighted images at baseline **(A,B)** and at the last follow-up evaluation **(C,D)** of the untreated patient (case 1). Panels **(A,C)** are planes crossing through the basal ganglia, while panels **(B,D)** are planes crossing through the corona radiata. The baseline study identifies capsular degeneration (arrows, **A**) and periventricular white matter injury with “tigroid appearance” (arrowheads, **B**) also known as the stripe sign. The tigroid appearance is also present in the white matter of the corona radiata (arrowheads, **B**). The extra axial spaces are enlarged. The follow-up examination, which is represented in panels **(C,D)**, shows rapid and diffuse deterioration of the neuroimaging findings both at the level of the capsular regions (arrows, **C**) and periventricular white matter (arrowheads, **C,D**). The volume loss of the brain parenchyma is not accompanied by a significant increase in caliber of the extra axial spaces.

Magnetic resonance imaging at 7 months showed diffuse periventricular T2/FLAIR hyperintensity, as well as subtle involvement of the basal ganglia and dentate nuclei bilaterally. Diffuse symmetric prominence of the subarachnoid space and ventricles indicated significant demyelination and brain atrophy (Figures 2A,B). At 5 years of age, his brain MRI showed extensive volume loss with ventriculomegaly, significant extra-axial fluid, and T2 signal abnormalities in the centrum semiovale (Figures 2C,D). There was significant thickening of the anterior Cauda Equina nerve roots on a spine MRI.

To evaluate disease progression in the peripheral nervous system, he underwent nerve conduction velocity testing at each evaluation. At the baseline evaluation, he had normal sural response. The median and peroneal sensory amplitude was normal, with abnormal distal latency and conduction velocity. The tibial motor nerves had normal amplitude and distal latency, but prolonged conduction velocity and F waves. At 5 years of age, the sural and median sensory responses were absent and the tibial, peroneal, and median motor amplitudes were decreased with prolonged latencies, conduction velocity and absent F waves.

He died at the age of 7 years from respiratory failure.

**FIGURE 3:**
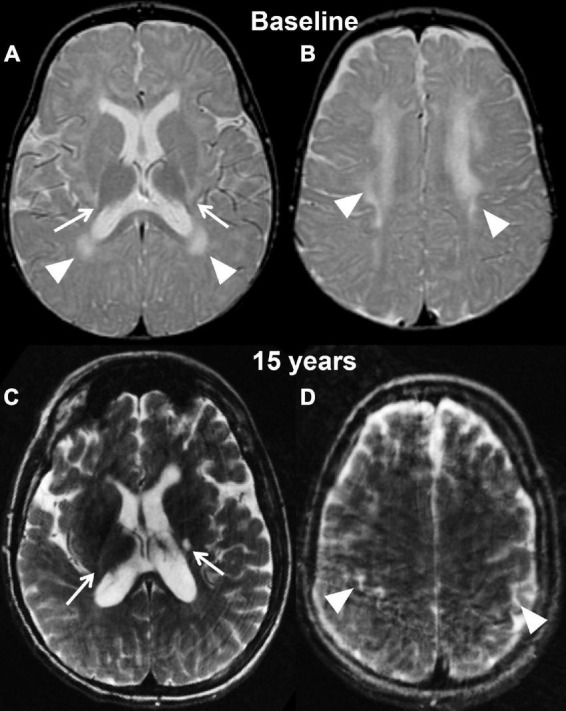
Axial T2 weighted images of case 2 at baseline pre-transplantation **(A,B)** and at the last follow-up evaluation **(C,D)**. Panels **(A,C)** are planes crossing through the basal ganglia, while panels **(B,D)** are planes crossing through the corona radiata. The baseline study shows the typical changes associated with Krabbe disease at the level of the capsular regions (arrows, **A**) and periventricular white matter and corona radiata (**A,B**, arrowheads) in the setting of a still unmyelinated brain. The follow-up evaluation demonstrates a normally myelinated brain, with the exception for the posterior limbs of the internal capsules (arrows, **A**). The white matter within the corona radiata is now normal appearing, with focal volume loss resulting in atrophy of the bilateral motor strip (arrowheads, **D**).

Using the newest classification system, patient 2, a female, was diagnosed with infantile Krabbe disease as a newborn due to a family history in an older sibling who died from disease progression at 12 months of age. Her mutation analysis showed homozygosity for the 30 kb deletion in the *GALC* gene, similar to case 1. During her first assessment (pre-transplant) she exhibited mild symptoms of disease such as arching of her back due to increased tone and feeding difficulties, placing her in stage II of disease progression (Escolar et al., 2006). Her pre-transplantation brain MRI at postnatal day 21 already showed bilateral and symmetric signal abnormalities in the areas of active myelination within the diencephalon and cerebrum, including within white matter tracts descending from the precentral gyrus (Figures 3A,B).

She was treated at 4 weeks of life with UCBT from a male donor with a 4/6 HLA match. She experienced no significant transplant-associated complications and successfully engrafted with 100% chimerism in peripheral blood.

Her disease progression post-UCBT was typical of transplanted patients in all developmental areas except gross motor (Escolar et al., 2006). She was never able to walk independently. Her motor disease during her teenage years rapidly worsened mostly due to peripheral neuropathy. However, her survival and developmental outcomes far exceeded those described in the natural history study of patients with similar disease onset (Beltran-Quintero et al., 2019). She was able to use a powered wheelchair for mobility and an augmentative communication device for communication. Her overall development progressed at a lower level compared to same-age peers. Cognitive skills were normal but as she became more motorically impaired, they became difficult to capture during standardized testing (Figure 1C). While initially within the normal range, at approximately 12 months her height’s percentile began to drop, but her growth curves showed normal head circumference (Figure 1D).

Her first post-transplant MRI scan at age 9 months showed a slight increase in overall brain volume and no evidence for disease progression. Subsequent scans demonstrated interval-decreased high T2 signal within bilateral periventricular regions and corona radiate at age 2 and 3, suggestive of progression of myelination. There was stable high T2 signal within the internal capsule. Findings remained stable at her 4-year follow-up scan. Her last brain MRI at 14 and 15 years (2 weeks before her death) showed mild atrophy with supratentorial and infratentorial volume loss and ex vacuo dilation of the ventricles, T2 prolongation involving the left posterior limb of internal capsule and thinning of the corpus callosum (Figures 3C,D). In addition, her lumbar spine demonstrated thickening of the Cauda Equina nerve roots.

She underwent nerve conduction velocity tests to evaluate disease progression in the peripheral nervous system. At 3 weeks of age, a sural sensory distal latency response was markedly abnormal, as well as amplitude and conduction velocity. Peroneal motor response was abnormal in latency, amplitude and conduction and F wave was non-obtainable. By 15 years, the sural and medial sensory response was absent and the peroneal motor response showed prolonged latency, low amplitude and markedly abnormal conduction that had progressed further when compared to previous year.

She required G-tube placement at 6 years due to problems keeping up with caloric intake and eating very slowly which started affecting her quality of life. At 9 years, she started having respiratory infections, and began to develop progressive scoliosis. By 13 years she had developed apnea during sleep, difficulties tolerating feeds and worsening of scoliosis. She eventually died at 15 years of age due to respiratory depression and cardiac arrest, severe scoliosis, dysautonomia and worsening of peripheral nerve disease.

### Pathology Results

#### Central Nervous System

**FIGURE 4:**
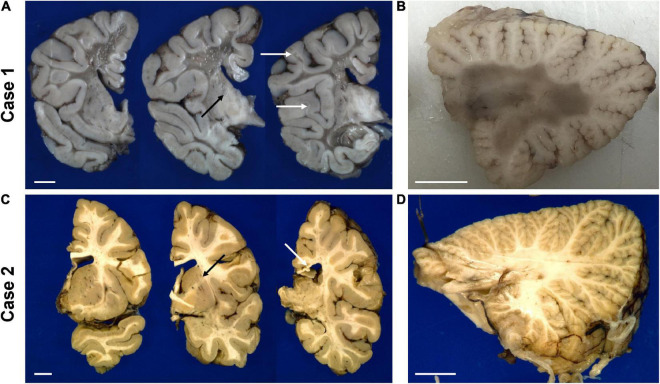
Representative gross photographs of coronal sections of the forebrain **(A,C)** and sagittal sections of the cerebellum **(B,D)** of the untreated case 1 (*top row*) and transplanted case 2 (*bottom row*). The white arrows in panel **(A)** indicate examples of residual white matter between preserved cortical ribbon and end-stage white matter. The white arrow in panel **(C)** points to thinned posterior corpus callosum. The black arrow in panels **(A,C)** indicate the internal capsule. The scale bar is set to 1 cm for each panel.

Gross examination of case 1 revealed an overall small brain weighing 894 g [expected 1,330 ± 10 g (Dekaban, 1978)] with marked white matter volume reduction and widespread near-complete myelin loss with minimal preservation of subcortical U-fibers (Figure 4A). While the cerebral cortical ribbon showed normal thickness and coloration, the cerebellar cortex exhibited atrophy and induration (Figure 4B).

**FIGURE 5:**
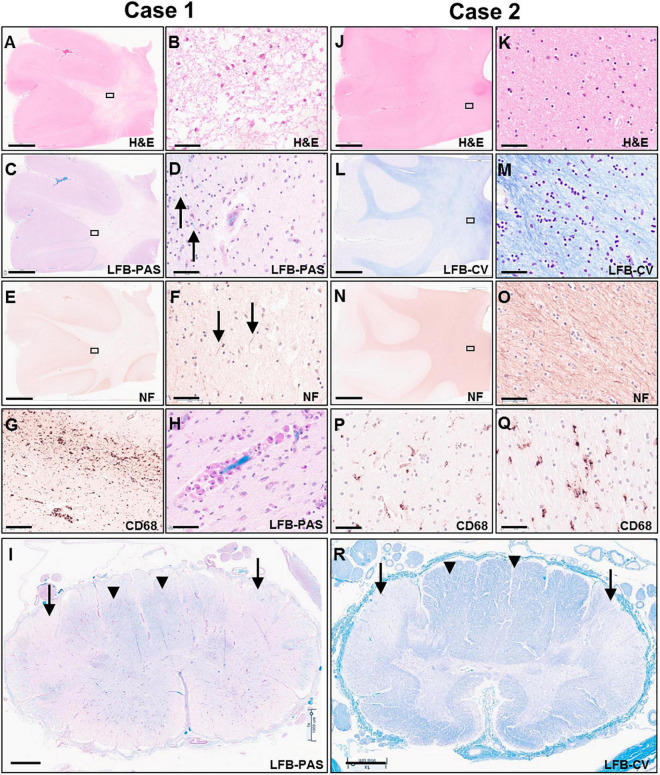
Photomicrographs of representative H&E **(A,B,J,K)**, LFB-PAS **(C,D,H,I)**, LFB-CV **(K,L,R)**, neurofilament **(E,F,N,O)** and CD68 **(G,P,Q)** stained sections of the untreated case 1 (left two columns) and transplanted case 2 (right two columns). The rectangular boxes in the low power images indicate the region shown in the adjacent higher-power image. Images **(A–H,J–Q)** show frontal lobe (middle frontal gyrus) and images **(I,R)** demonstrate thoracic spinal cord. Arrows in images **(D,F)** indicate rare residual axons in case 1. Arrows in panels **(I,R)** point to the lateral corticospinal tracts, whereas arrow heads indicate posterior columns. Scale bars are set to 5 mm in panels **(A,C,E,J,L,N)**; to 1 mm in panels **(I,R)**; and to 50 μm in panels **(B,D,F,G,H,K,M,O,P,Q)**.

Microscopic examination of H&E and LFB stained sections confirmed the presence of predominantly end-stage white matter rarefaction in centrum semiovale, corpus callosum, internal capsule, cerebellum, and corticospinal tracts in the brainstem. The severe to essentially complete myelin loss and fibrillary gliosis were accompanied by severe oligodendrocyte depletion and near-complete absence of neurofilament-positive axons (Figures 5A–F). Only focal white matter areas in the centrum semiovale displayed slightly more active disease with reactive astrocytes and perivascular foamy macrophage clusters, but classic globoid cells were rare, and myelin was similarly depleted in these areas as in end-stage areas (Figures 5G,H). The severe involvement of corticospinal tracts extended into the spinal cord with gliosis, rarefaction and near-complete myelin depletion but without macrophage or globoid cell infiltrates (Figure 5I). Moderate to severe myelin loss was also evident in all other fiber tracts. Posterior columns showed more severe loss at lumbar than cervicothoracic levels.

**FIGURE 6:**
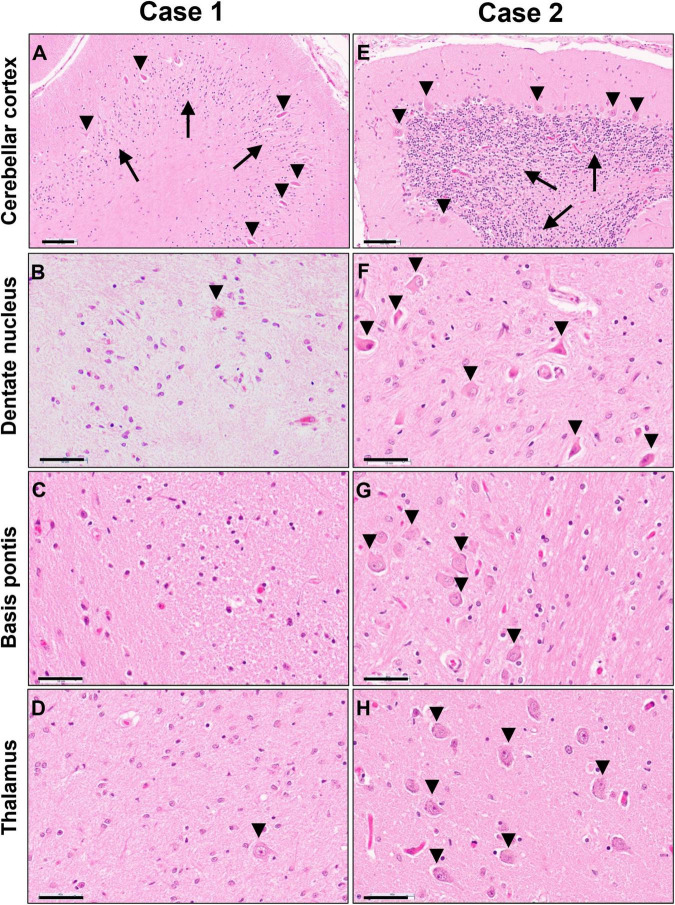
Photomicrographs of H&E stained sections of cerebellar cortex **(A,E)**, cerebellar dentate nucleus **(B,F)**, basis pontis **(C,G)** and thalamus **(D,H)** of the untreated case 1 (left column) and transplanted case 2 (right column). Arrows in panels **(A,E)** indicate the granular cell layer and arrowheads point to Purkinje neurons. Arrowheads in panels **(B,D,F,G,H)** indicate neuronal cell nuclei, illustrating the severe neuronal loss in the untreated case. Scale bars are set to 100 μm in panels **(A,E)** and to 50 μm in all other images.

Gray matter regions exhibited highly varied involvement. While the cerebral cortex showed only mild gliosis at the gray-white junction and patchy superficial and mid-laminar neuropil vacuolization without overt neuronal drop-out, the cerebellar cortex exhibited moderate to severe loss of granular neurons and highly variable, mild to severe loss of Purkinje neurons and associated Bergmann gliosis (Figure 6A). A few scattered Purkinje neurons displayed axonal torpedoes, and a few had a prominent swollen dendritic tree (meganeurites) in the molecular layer. Severe neuronal loss and gliosis were present in the cerebellar dentate nucleus, claustrum, in large parts of the thalamus and basis pontis (Figures 6B–D); moderate neuronal loss was present in inferior olives and minimal to moderate in spinal cord anterior horns.

The transplanted brain of case 2 also demonstrated reduced brain weight at 1,100 g, but only slightly less than expected for age and gender [1,280 ± 40 g (Dekaban, 1978)]. Unlike the non-treated brain, the white matter of the centrum semiovale and cerebellum appeared well myelinated on gross examination and of nearly normal volume with only mildly dilated lateral ventricles (Figures 4C,D). There was mild thinning of the posterior corpus callosum, and mild volume loss of cerebral peduncles and pyramids in the brainstem.

On microscopic examination, the white matter of the centrum semiovale and cerebellum exhibited variable pallor and moderate to severe myelin loss, with better preservation in periventricular regions but no clear demarcation of subcortical fibers. This was associated with variable, minimal to moderate loss of neurofilament-positive axons, which followed a similar pattern as the myelin depletion (Figures 5J–O). Mild diffuse gliosis and microglial activation with occasional small perivascular macrophage clusters was present. The microglial morphologies ranged from ramified to ameboid, but no globoid cells were identified (Figures 5P,Q). Oligodendroglial cells were readily identified with a possible subtle decrease in density in the paler white matter areas. The corticospinal tract was the most severely affected longitudinal fiber tract, with moderate to severe myelin loss extending from internal capsule through brainstem into spinal cord (Figure 5R), accompanied by marked loss of neurofilament-positive axons. Other spinal cord fiber tracts were better preserved but still showed mild to moderate myelin loss and at least mild axonal loss.

Gray matter regions were largely preserved with only a few exceptions. The cerebellar cortex demonstrated mild Bergmann gliosis with only focal minimal Purkinje cell loss (Figure 6E), but numerous Purkinje cell torpedoes were apparent in both vermis and lateral hemispheres. There appeared to be minimal to mild thinning of the granular cell layer. The dentate nucleus displayed marked hypercellularity due to reactive gliosis but without overt neuronal loss (Figure 6F). Similar but milder changes were noted in the inferior olives. Mild neuronal loss and gliosis were seen in the claustrum, and minimal to moderate involvement of the thalamus was present (Figure 6H). No overt neuronal loss was observed in cerebral cortex, basal ganglia, hippocampus, basis pontis, or spinal cord (Figure 6G).

#### Peripheral Nervous System

The findings from peripheral nerve examinations have been previously reported (Weinstock et al., 2020). In short, both cases showed variable myelin loss, axon loss, endoneurial fibrosis and patchy edema. While the non-transplanted nerve sections demonstrated a patchy infiltrate of endoneurial and subperineurial CD68 positive macrophages, only a few scattered macrophages were noted in the transplanted nerve samples. Ultrastructural examination by electron microscopy of peripheral nerve sections demonstrated the classic inclusions associated with Krabbe’s disease in the form of straight and slightly curved, cleft-like structures in the cytoplasm of Schwann cells, but this was not demonstrated in macrophages of the transplanted patient.

#### Detection of Donor-Derived Cells in the Transplanted Krabbe Patient

**FIGURE 7:**
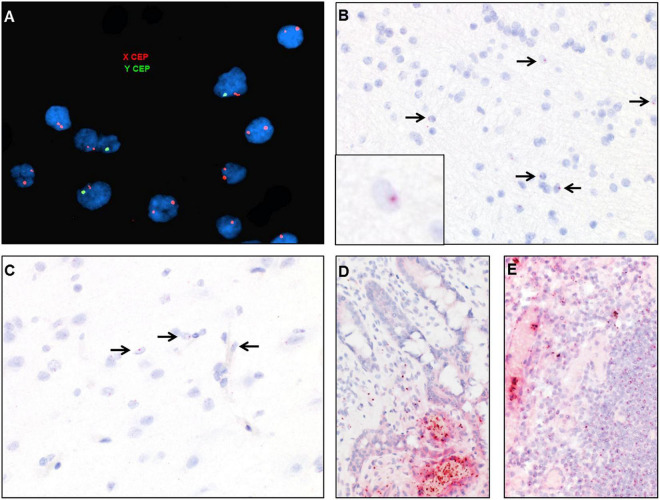
Identification of donor-derived male cells by DNA *in situ* hybridization with X (red) and Y (green) centromere probes of the white matter of case 2 **(A)** and by RNA *in situ* hybridization with Y-specific probes (red) and hematoxylin counterstain of cortical white matter **(B)**, cortical gray matter **(C)**, small bowel **(D)**, and lymph node **(E)**. Arrows point to scattered cells with small dot-like positive signal.

DNA fluorescent *in situ* hybridization (FISH) studies with X and Y chromosome probes revealed that the majority of cells in the brain had a female XX karyotype with a scattered admixed population of cells with a male XY karyotype (Figure 7A). These findings were confirmed by chromogenic RNA *in situ* hybridization (ISH) using a cocktail probe for three genes located on the Y chromosome. Scattered positive cells of presumed donor origin were identified throughout the frontal white matter in parenchymal and perivascular locations, accounting for about 4.8% of the total cell population (Figure 7B). Scattered positive cells were also present in the overlying cortex (Figure 7C). However, no definitive positive cells were identified in the spinal cord or peripheral nerve, possibly due to the overall low number of infiltrating macrophages. RNA ISH studies on small bowel and lymph node samples demonstrated that the majority of lymphocytes and other hematopoietic cells were of donor origin (Figures 7D,E).

## Discussion

Hematopoietic stem cell transplantation is currently the only available treatment modality for Krabbe patients. Most survivors show durable engraftment of donor-derived cells with normalization of peripheral blood galactocerebrosidase levels. If performed in the pre-clinical stage, transplantation has been shown to improve lifespan and functional abilities, although some children experience delays in expressive language and gross motor skills (Krivit et al., 1998; Escolar et al., 2005; Sakai, 2009; Wright et al., 2017; Allewelt et al., 2018). These functional improvements are accompanied by progressive myelination on serial brain MRI scans, as also observed in our transplanted case.

We now describe for the first time the neuropathological findings in a transplanted Krabbe disease case. Compared to our untreated case and other infantile cases reported in the literature (Wenger et al., 2001), the transplanted brain demonstrated a markedly improved degree of myelination, albeit not reaching the levels expected for a normal brain. The myelination abnormalities were most prominent in centrum semiovale, cerebellar white matter and corticospinal tracts, areas typically affected early and severely in Krabbe disease. With this patient being already mildly symptomatic at birth, it is likely that these areas were already involved pre-transplant, as confirmed by early postnatal MRI findings. The decreased degree of myelination is most likely a reflection of injury that occurred early in the disease course as no evidence of post-transplant active demyelination was seen in the central nervous system. This anatomic distribution also correlated well with the patient’s severe clinical deficits in gross motor skills.

Data from twitcher mice indicate that it takes several weeks after transplantation until GalC activity increases in the brain, psychosine levels reduce and pathologic features improve (Yeager et al., 1984; Ichioka et al., 1987; Hoogerbrugge et al., 1988a, b; Suzuki et al., 1988). Thus, most if not all of the CNS myelin loss seen in our case likely happened before or in the early weeks after transplantation when there is a period of conditioning and cell engraftment during which the disease continues to progress. The lack of active demyelination later in life is consistent with serial imaging studies which demonstrated stable white matter abnormalities. The decrease in myelin was accompanied by concurrent axonal loss, which likely represents a major factor in limiting complete remyelination and restoration of white matter integrity. While we do not have data on brain GalC activity or psychosine levels in our autopsy cases, the absence of globoid cells and lack of active demyelination highly suggest that the enzymatic defect was significantly ameliorated by the transplant procedure, at least in the central nervous system.

In addition to the beneficial effects of UCBT on myelination, we also noted significant preservation of neuronal cell populations. While Krabbe disease is mainly considered a white matter disease, many untreated cases show significant neuronal depletion in a stereotypical distribution pattern involving primarily cerebellar cortex, dentate nucleus, inferior olives, basis pontis, thalamus, and claustrum (Kofler et al., 2019). While these regions showed marked neuronal loss in our untreated case, only subtle changes were present after transplantation. The mechanisms underlying neuronal loss in Krabbe disease, and in particular the selective vulnerability of certain cell populations, are poorly understood. There is some evidence from twitcher mice that neurons do accumulate psychosine, albeit less than oligodendrocytes, and that psychosine has neurotoxic effects in culture (Castelvetri et al., 2011). *In vivo*, axonopathy is observed early in twitcher mice, before the onset of demyelination, but neuronal loss is not seen until late stages (Castelvetri et al., 2011). Our findings suggest that the time course for neuronal loss may be different in human Krabbe brains, as mild neuronal loss was present in stereotypical brain regions in the transplanted case. We assume that this neuronal injury occurred in the pre- or early post-transplantation period, similar to the white matter injury. The lack of more advanced neuronal loss suggests that transplantation was effective in halting further neurodegeneration by either cross-correcting neuronal enzyme deficits or by otherwise preventing injury cascades leading to neuronal death.

Unlike the stabilization observed clinically, radiologically and pathologically for the CNS disease, many transplanted Krabbe patients experience minimal clinical benefit in the peripheral nervous system. This impression is supported by serial nerve conduction studies which demonstrated only transient improvements of nerve conduction velocities after UCBT (Wright et al., 2017). While postmortem examination identified only minimal macrophage infiltration into peripheral nerves, less than seen in non-transplanted cases, significant nerve pathology was present and characterized by myelin loss, axon loss, endoneurial fibrosis, subperineurial, and endoneurial edema, and ultrastructural inclusions in Schwann cells. These results share many similarities with the peripheral nerve findings in transplanted twitcher mice. After a lag period of several weeks, the mice showed increased numbers of remyelinated fibers, disappearance of the typical inclusion-laden macrophages and absence of active demyelination, but persistence of inclusions in Schwann cells and continued presence of demyelinated fibers (Kondo et al., 1988). More recent experiments with GalC-deficient Schwann cells demonstrate that these cells are inefficient in receiving GalC from surrounding cells and are not appropriately cross-corrected (Weinstock et al., 2020). These data suggest that GalC-competent, donor-derived macrophages may provide some benefit in peripheral nerves through immunomodulatory mechanisms (Reddy et al., 2011; Karumuthil-Melethil and Gray, 2016; Potter and Petryniak, 2016; Weinstock et al., 2020), but are unable to correct the metabolic defect in Schwann cells leading to progressive PNS disease in transplant recipients.

Like most UCBT survivors, our transplanted case showed durable engraftment of donor-derived cells with normalization of peripheral blood galactocerebrosidase levels. This was confirmed by postmortem *in situ* hybridization studies demonstrating an abundance of male donor-derived hematopoietic cells in spleen and gastrointestinal tract of our female recipient. Furthermore, male cells were identified in the central nervous system, comprising about 5% of the total cell population in the white matter. The cells appeared scattered throughout the parenchyma and not restricted to intra- or perivascular spaces. These findings suggest that long-term integration of donor-derived cells into the CNS occurred.

While technical limitations prevented us from further characterizing the lineage of these donor-derived cells, they most likely represent monocyte-derived microglia/macrophages, an interpretation consistent with morphological features of these cells and limited evidence in the literature. In twitcher mice, donor-derived macrophages were observed in the CNS after bone marrow transplantation, but not until several weeks after the procedure. These donor-derived cells were seen most frequently in areas affected by the disease, and the time course of infiltration aligned with a gradual increase in GalC activity in the brain, suggesting that the donor-derived cells were the source for the improved enzyme activity (Hoogerbrugge et al., 1988b). In a small case series of pediatric and young adult female patients receiving bone marrow transplants for hematological disorders, male donor-derived cells were identified in intravascular, perivascular, and intraparenchymal locations in postmortem brain tissue sections and corresponded in distribution to leukocyte common antigen (LCA)-positive mononuclear leukocytes (Unger et al., 1993).

While these early studies suggested that donor-derived myeloid cells engrafted into the brain, it remained unclear if they truly differentiated phenotypically into microglial cells. In a mouse model of Gaucher disease, a small subset of donor-derived cells appeared consistent with parenchymal microglia and displayed ramified morphology (Krall et al., 1994). In a single case report of a female patient who underwent UCBT for lymphoma, donor-derived cells were identified in the brain using immunohistochemical staining against a mismatched HLA antigen. Many of the positive cells adopted a ramified morphology and co-labeled with microglial marker Iba1 but not with astrocyte marker GFAP (Takahashi et al., 2015). An autopsy study of two transplanted metachromatic leukodystrophy (MLD) cases found metabolically competent donor macrophages throughout the white matter expressing arylsulfatase A, the deficient enzyme in MLD (Wolf et al., 2020). Taken together, these results suggest that donor-derived myeloid cells are capable of entering and durably engrafting into the CNS after BMT or UBCT. Moreover, they resemble microglial cells, at least morphologically, and are functionally competent in a manner to supply sufficient enzyme activity to halt disease progression in Krabbe disease. Further studies are needed, though, to determine if these donor-derived cells are phenotypically similar to host microglia or represent a unique phenotype. Recent evidence supports the latter, by finding that brain-engrafting macrophages have a transcriptional profile distinct from microglia (Cronk et al., 2018). In the brains of MLD patients, increased macrophage expression of mannose receptor was seen in transplanted compared to non-treated patients, suggestive of skewing toward an alternative activation state, which is considered to be anti-inflammatory and supportive of oligodendrocyte survival and remyelination (Wolf et al., 2020).

In conclusion, our data provide evidence that UCBT has beneficial effects in pre-clinical Krabbe disease by leading to engraftment of donor-derived cells into the brain and halting progressive demyelination and neuronal loss. While this treatment leads to stabilization of CNS disease, both clinical and pathological findings indicate progression of PNS disease, resulting in a markedly changed disease phenotype in long-term transplant survivors. The reasons for these differences in efficacy remain under investigation, but a leading hypothesis is the limited degree of Schwann cell cross-correction as demonstrated in a recent publication (Weinstock et al., 2020). Adeno-associated virus (AAV) gene therapy, alone or in combination with transplantation, has led to improved outcomes in murine and canine Krabbe disease models (Reddy et al., 2011; Rafi et al., 2015; Bradbury et al., 2018). Combination of UCBT with gene therapy has been shown to have a synergistic effect with better outcomes than each therapy alone. This combination approach is now in human phase I/II clinical trials, in the hope that this two-pronged approach can overcome the limitations of stand-alone UCBT/BMT.

## Data Availability Statement

The raw data supporting the conclusions of this article will be made available by the authors, without undue reservation.

## Ethics Statement

The studies involving human participants were reviewed and approved by the Human Research Protection Office at the University of Pittsburgh and the Office of Human Research Ethics at the University of North Carolina - Chapel Hill. Written informed consent was obtained from the minor(s)’ legal guardian/next of kin for the publication of any potentially identifiable images or data included in this article.

## Author Contributions

JK: conceived, designed and performed the pathology analyses, and drafted the manuscript. MB-Q: collected the clinical data and drafted the clinical section. AR: contributed to the clinical data and coordinated the NDRD autopsy program. GZ: contributed to the radiology data. SK: collected the clinical data. ME: conceived and designed the study, managed the patients, and performed the clinical examinations. All authors reviewed and revised the manuscript and approved the final version.

## Conflict of Interest

ME had financial equity and employment at Forge Biologics. The remaining authors declare that the research was conducted in the absence of any commercial or financial relationships that could be construed as a potential conflict of interest.

## Publisher’s Note

All claims expressed in this article are solely those of the authors and do not necessarily represent those of their affiliated organizations, or those of the publisher, the editors and the reviewers. Any product that may be evaluated in this article, or claim that may be made by its manufacturer, is not guaranteed or endorsed by the publisher.
